# Economic Burden of ICU-Hospitalized COVID-19 Patients: A Systematic Review and Meta-Analysis

**DOI:** 10.7759/cureus.41802

**Published:** 2023-07-13

**Authors:** Fotios Tatsis, Elena Dragioti, Mary Gouva, Vasilios Koulouras

**Affiliations:** 1 Faculty of Medicine, School of Health Sciences, University of Ioannina, Ioannina, GRC; 2 Research Laboratory Psychology of Patients, Families & Health Professionals, Department of Nursing, School of Health Sciences, University of Ioannina, Ioannina, GRC; 3 Pain and Rehabilitation Centre, and Department of Health, Medicine and Caring Sciences, Linköping University, Linköping, SWE; 4 Department of Intensive Care Unit, University Hospital of Ioannina, Ioannina, GRC

**Keywords:** pandemic, policy, icu, covid-19, health economics

## Abstract

The impact of the coronavirus disease 2019 (COVID-19) pandemic on the global economy is far-reaching and difficult to assess accurately. We aimed to systematically determine the magnitude of the costs and the economic burden of intensive care for hospitalized COVID-19 patients since the onset of the pandemic by means of a systematic review. We conducted a PRISMA 2020-compliant (protocol: PROSPERO CRD42022348741) systematic review by searching PubMed, EMBASE, and Web of Science for relevant literature. We included studies that presented costs based on a primary partial economic evaluation. Using the Consolidated Health Economic Evaluation Reporting Standards checklist and the population, intervention, control, and outcome criteria, we established the risk of bias in studies at the individual level. Daily cost per ICU admission and total cost per ICU patient of the original studies extracted. A random effect model was adopted for meta-analysis whenever possible. Of the 1,635 unique records identified, 14 studies related to ICU-hospitalized costs due to COVID-19 were eligible for inclusion. Included studies represented 93,721 hospitalized COVID-19 patients. Regarding total direct medical costs, the lowest cost per patient at ICU was observed in Turkey ($2,984.78 ± 2,395.93), while the highest was in Portugal ($51,358.52 ± 30,150.38). The Republic of Korea reported the highest length of stay of 29.4 days (±17.80), and the lowest is observed in India for nine days (±5.98). Our findings emphasize COVID-19's significance on health-economic outcomes. Limited research exists on the economic burden of COVID-19 in the ICU. Further studies on cost estimates can enhance data clarity, enabling informed analysis of healthcare costs and aiding efficient patient care organization by care providers and policymakers.

## Introduction and background

The novel coronavirus disease, currently known as coronavirus disease 2019 (COVID-19; caused by the SARS-CoV-2 pathogen), was first introduced as a pandemic in December 2019 in Wuhan, China's Hubei province [1]. The outbreak of COVID-19 has created a serious global health threat with extraordinary implications for health, society, and the global economy [2,3]. As a result of the COVID-19 pandemic, the global economy is facing a variety of impacts beyond the global health burden that are difficult to assess accurately. It is estimated that the pandemic has reduced global economic growth. Current economic forecasts reflect the risks to a sustained global recovery posed by geopolitical developments, potential monetary policy changes by central banks, a resurgence of infectious COVID-19 cases, inflationary pressures related to supply chain and labor market issues, and pent-up consumer demand. Meanwhile, supply shortages reflect ongoing disruptions in labor markets, manufacturing and supply chain bottlenecks, and shipping and transportation restrictions [4].

The demands on the healthcare system and the critical resource shortages created (e.g., hospital beds, intensive care unit (ICU) beds, ventilators, and staff) have also exacerbated [5]. Healthcare systems and hospitals around the world have adapted and reorganized to meet the challenges of this pandemic. In the first two years of the pandemic radical changes were focused on increasing bed capacity in intensive care units, developing COVID-19 wards to isolate and treat infected patients, and establishing field hospitals [6]. From an economic perspective, the spread of COVID-19, the ever-increasing number of patients, and the complications of the disease have imposed high direct medical and indirect costs on patients, the healthcare systems, and governments [7]. As far as the economic burden of direct medical costs is concerned, although the costs vary with the number of infected people, the severity of the disease, the mean length of stay in the hospital, and other factors [8,9]. Studies showed that the medical costs of patients with COVID-19 were significantly higher than those of other infectious diseases due to the higher likelihood of hospitalization and mortality [10]. These circumstances are also right about the need for COVID-19 patients for special care services and the related costs [11-13]. As with any new disease, cost data related to the burden of COVID-19 has been scarce. Several studies on the medical costs of COVID-19 in the intensive care unit have recently been published in different countries. Because of this increase in the number of research studies and their long-term economic impact on healthcare budgets, understanding and summarizing the current evidence is critical to informing governments of the economic burden of COVID-19.

Because knowing the financial impact of the COVID-19 pandemic on the health system is essential to inform and support policymakers on possible adjustments of budgets dedicated to health systems and, in particular, to hospitals. The aim of this study was to systematically determine the magnitude of the costs and the economic burden of intensive care for hospitalized COVID-19 patients since the onset of the pandemic.

## Review

Materials and Methods

Study Design and Registration

We conducted this systematic review following the Preferred Reporting Items for Systematic Reviews and Meta-Analyses (PRISMA) guidelines [14,15]. The study protocol was a priori registered in the International Prospective Register of Systematic Reviews database (PROSPERO: CRD42022348741).

Search Strategy

Two researchers (FT, ED) independently searched PubMed, EMBASE, and the Web of Science up to September 30, 2022. Relevant literature was searched by using combinations of three categories of keywords: 1) COVID-19, 2) cost, and 3) ICU. The terms have been translated into the query language of the respective database. For each relevant study identified during the search, we also manually scooped the cited references to collect additional eligible studies that we might have missed in the electronic search.

Selection Criteria

The study question of this systematic review was specified using the Population, Exposure, Outcomes (PEO) framework. The population of interest included patients hospitalized exclusively in ICUs, the exposure considered patients with COVID-19, and the main outcomes were the daily cost per ICU admission and the total cost per ICU patient, as reported in the original studies. Secondary outcomes were length of stay in the ICU and mean cost per patient.

Our selection was limited to studies that estimated costs based on a primary partial economic evaluation, such as cost-of-illness (COI) studies, cost analysis, observational reports (cross-sectional studies, and prospective and retrospective cohorts). We included every single study that presented at least one cost outcome in the ICU. Letters, reviews, commentaries, editorials, case reports, case series, and papers without sufficient information to clearly identify methods, sources, or unit costs were excluded. We also excluded studies that estimated outcomes using predictive or prognostic models.

Data Collection

All citations obtained using the search strategy were imported into the reference management software package EndNote (Clarivate, London United Kingdom), and duplicates were deleted based on an exact match of author, year, title, and abstract. Two reviewers independently screened the records based on title and abstract reading. After excluding those that were deemed irrelevant, the full texts of the remaining records were further assessed for inclusion. Any discrepancies were resolved through discussion with a third senior reviewer.

Data Extraction

One reviewer extracted all the data from eligible studies which were double-checked by a second reviewer, and any disagreements were also discussed with a third senior reviewer. The following data were extracted: author, year of publication, study period, population parameters, reported economic evaluation outcomes, ICU cost per day, cost per ICU admission, and length of stay. Costs extracted were converted into 2021 US Dollars (US$ 2021) using the Purchasing Power Parity (PPP)-adjusted proposed on the cost converter tool from CCEMG-EPPI Centre [16]. To further facilitate the comparison of costs across countries, the costs were compared with the country's gross domestic product (GDP). GDP was obtained from the World Bank [17] in US$. Whenever feasible, general ward cost data were also recorded.

Study Quality Assessment

The quality assessment per included study was evaluated by two independent reviewers according to the Consolidated Health Economic Evaluation Reporting Standards (CHEERS) guideline against 24 checklist items [18]. To obtain an overall quality assessment, the studies scored one point for each fully met criterion, 0.5 for each partially met, and 0 for each criterion if no or only limited information was reported. A percentage of points is then formed, in which all criteria are weighted equally (criteria that do not apply were excluded from the calculation). Studies with a score of 75% or greater are considered high quality, scores in the range of 50-74% are considered moderate, and scores below 50% are considered poor reporting quality. In case of disagreement in the assessment, consensus was reached through discussions with the research team.

Data Synthesis

We performed a quantitative synthesis of the included data and presented them in comprehensive tables based on our PEO framework. Descriptive summary of the findings from the included studies such as study settings, country in which the study was conducted, the perspective of the studies, type of economic assessment performed and analytical approach used in the studies, category of reported costs, type of interventions and outcomes, etc. were carried out.

If a study reported sample size, mean, and standard deviation (SD), we calculated the 95% confidence interval (CI) using the expression: $CI = \bar{x} \pm z \frac{s}{\sqrt{n}}$, where $CI$ = confidence interval; $\bar{x}$ = sample mean; $z$ = confidence level value; ​​​​​​​$s$ = sample standard deviation; and ​​​​​​​$n$ = sample size.

Additionally, if any study reported sample size, interquartile range (IQR), and the median of the sample, we calculated the mean of the sample [19] and standard deviation of the sample [20]. In two studies [21,22], the IQR reported by one value as the difference between the third and first quartile, and one study reported only the median value without IQR. A random effects meta-analysis with Der Simonian and Laird variance estimator was employed [23], whenever possible, to compare the ICU costs versus the general ward costs. The I^2^ statistic was computed to evaluate inconsistency (I^2^>50% indicated high inconsistency) between studies [24]. The difference in raw means between the ward and the ICU was used in synthesizing studies' results. This is because the raw mean difference could easily be translated into an actual comparison measurement [25]. All analyses were conducted in Stata/SE, version 17.0 (StataCorp LLC, College Station, Texas).

Results

Search Results

**Figure 1:**
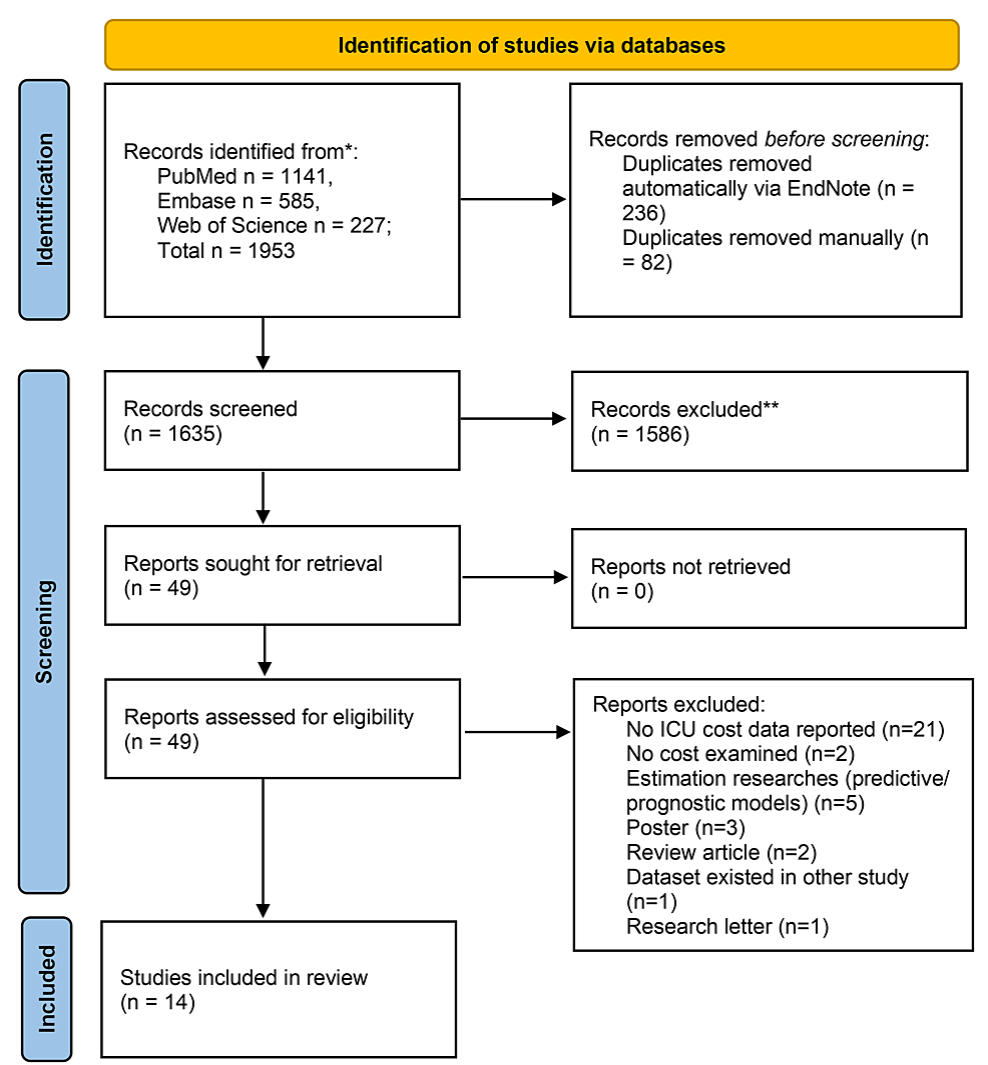
Study selection flowchart * indicates studies reviewed; ** indicates studies excluded

A total of 1,953 potentially relevant records were identified through the electronic database search. After the removal of duplicates, 1,635 unique records were obtained and screened. Altogether, 49 full-text articles were checked for eligibility, and 14 individual publications were eventually included in this systematic review (Figure 1) [21,22,26-37]. Most of the publications (n=21) were excluded because they did not report ICU costs.

Characteristics of Included Studies

Table 1 summarizes the general characteristics of the studies included in this systematic review. The eligible studies were published between 2020 and 2022 and included a total of 93,721 patients admitted to ICU, ranging from 29 to 88,590. Countries analyzed in the individual studies included three studies from Turkey and one study from the following countries: Colombia, Spain, Canada, Iran, the Republic of Korea, Saudi Arabia, Brazil, the United States, Romania, India, and Portugal. All studies discussed the direct medical costs of ICU hospitalizations for COVID-19 and examined at least one cost in ICU due to COVID, either total cost per patient or cost per day per patient. Some studies reported mean cost with SD, while others reported the median cost with IQR. Where possible, missing costs were calculated, along with the 95% CI.

**Table 1:** Summary of included studies N/R = not reported, N = total number of patients admitted to ICU, ICU = intensive care unit, LOS = length of stay, SD = standard deviation, CI = confidence interval, IQR = interquartile range, currency = US$

Study, year	Country	Study design	Period	N	LOS ICU					ICU daily cost per patient					ICU cost per patient				
					mean	SD	median	IQR	95% CI	Median	IQR	Mean	SD	95% CI	Mean	SD	95% CI	Median	IQR
Kavalci, 2021 [21]	Turkey	Retrospective study	N/R	71			11.50	11.00										14,875.83	15,814.19
Oksuz et al., 2021 [22]	Turkey	Retrospective cohort study	From March 11, 2020, to July 31, 2020	163	14.8	12.00	13	12.00	13						4,131.94	4,298.47	3,470 - 4,790	2,859.19	3,831.29
Alvis-Zakzuk et al., 2022 [26]	Colombia	Retrospective cost-of-illness study	From March 15, 2020, to May 29, 2020	34	10.2	7.20			7.78						3,945.05	2,675.02	3,050 - 4,840	4,203.60	2,112.01 - 5,568.39
Calderón-Moreno et al., 2022 [27]	Spain	Retrospective cohort study	From the beginning of the pandemic to June 30, 2020	283											22,940.63				
Cheung et al., 2022 [28]	Canada	Retrospective matched-cohort study	From January 1, 2020, to June 30, 2020	944											48,018.40	31,434.29	46,000 - 50,000		
Darab et al., 2021 [29]	Iran	Partial economic evaluation and cross-sectional cost-description study	From March 2020 to July 2020	36											10,552.41	7,645.00	8,050 - 13,100		
Gedik, 2020 [30]	Turkey	Cost analysis	From March 17, 2020, to May 11, 2020	66	14.74	13.19			11.6			236.72	94.45	214 - 260	2,984.78	2,395.93	2,410 - 3,560		
Jang et al., 2021 [31]	Republic of Korea	N/R	From the beginning of the pandemic to May 15, 2020	846	29.4	17.80			28.2						12,222.89	11,729.86	11,400 - 13,000		
Khan et al., 2020 [32]	Saudi Arabia	N/R	From March 1 to May 29, 2020	222								6,724.52							
Miethke-Morais et al., 2021 [33]	Brazil	Partial economic evaluation (cost of illness) and a prospective, observational cohort study	From March 30 to June 30, 2020	1,683								62,066.28							
Ohsfeldt et al., 2021 [34]	USA	Observational study	From April 1, 2020, to December 31, 2020	89,132	7.7	9.1	5		7.64	2,962.32		3,660.34	3,355.94	3,630 - 3,690	27,170.98	35,130.56	26,900 - 27,400	13,722.43	
Popescu et al., 2022 [35]	Romania	N/R	From September 1, 2021, to October 31, 2021	36	14.1	8.1			11.4	311.34	129.60 - 674.66				7,808.09	12,254.56	3,810 - 11,800	5,368.89	754.42 - 16,367.85
Reddy et al., 2021 [36]	India	Retrospective direct medical care cost analysis	From June 2020 to December 2020	176	9	5.98	9	5.00 - 13.00	8.12	889.66	674.08 - 1105.59	889.79	322.54	842 - 937	12,693.59			10,907.50	6,885.23 - 16,626.61
Seringa et al., 2022 [37]	Portugal	Observational study	From March 1, 2020, to May 31, 2020	29	10.1	6.00	9	7.00 - 12.00	7.92						51,358.52	30,150.38	40,400 - 62,400	46,414.03	31,179.09 - 62,866.68

The study designs across the studies were as follows: retrospective cost-of-illness study [26], retrospective cohort study [22,27], retrospective matched-cohort study [28], partial economic evaluation and cross-sectional cost-description study [29], cost analysis [30], retrospective study [21], partial economic evaluation (cost of illness) and prospective, observational cohort study [33], observational study [34,37], retrospective direct medical care cost analysis [36]. Three studies [31,32,35] did not specify their study design.

Clinical patient data were obtained from either hospital databases [21,22,26,27,29,33,35-37] or from larger national databases such as the Ontario Laboratory Information System COVID-19 database [28], Social Security Institution of the Republic of Turkey [30], Health Insurance Review & Assessment Service of Korea [31], Health Electronic Surveillance Network (HESN) database of the Saudi Ministry of Health [32] and the US Premier Healthcare Database [34]. The same databases were utilized for patient management costs, as most hospitals stored both clinical and financial data, with a few exceptions. In Spain and Portugal, the costs of patient management in public hospitals were mandated to be published by law [27, 37], while in Turkey, one study [22] reported that costs were retrieved from the Social Security Institution's Health Implementation Notification.

Methodological Quality of Included Studies

Details of the Consolidated Health Economic Evaluation Reporting Standards (CHEERS) results per domain are presented in Table 2. The average quality score for all studies was 64%. The maximum and minimum quality scores were 76% [33] and 36% [27], respectively. Two of the studies were rated as excellent quality, scoring 75 or higher [33, 36], while ten studies were classified as moderate quality, scoring within the range of 50-74 [22,26,28-32,34,35,37]. Two studies were considered to have low-quality scores of less than 50% [21,27]. We observed relatively low-quality indicators, which can be attributed to the fact that the original studies did not adhere to a specific form of economic analysis, such as cost-effectiveness analysis, and did not necessitate the use of any decision analytical model.

**Table 2:** CHEERS evaluation NA = not available, CHEERS = Consolidated Health Economic Evaluation Reporting Standards

Study	Title	Abstract	Background and objectives	Target population and subgroups	Setting and location	Study perspective	Comparators	Time horizon	Discount rate	Choice of health outcomes	Measurement of effectiveness	Measurement and valuation of preference-based outcomes	Estimating resource use and costs	Currency, price data and conversion	Choice of model	Assumptions	Analytical methods	Study parameters	Incremental costs and outcomes	Characterizing uncertainty	Characterizing heterogeneity	Study findings, limitations, generalizability and fit with current knowledge	Source of funding	Conflict of interest	Score
Kavalci, 2021 [21]	0	0	0	1	1	0	1	1	0	0	0	NA	0	0.5	NA	NA	0	1	0.5	0	0	1	0	1	38%
Oksuz et al., 2021 [22]	1	1	1	1	1	1	1	1	0	0	0	NA	0	1	NA	NA	0	1	1	0	1	1	1	1	71%
Alvis-Zakzuk et al., 2022 [26]	0.5	1	1	1	1	1	1	1	0	0	0	NA	0	1	NA	NA	0	1	1	0	1	1	1	1	69%
Calderón-Moreno et al., 2022 [27]	1	1	1	1	1	1	1	0	0	0	0	NA	0	0.5	NA	NA	0	0	0	0	0	0	0	0	36%
Cheung et al., 2022 [28]	0.5	1	1	1	1	1	1	1	0	0.5	0	NA	0	1	NA	NA	0	1	1	0	1	1	1	1	71%
Darab et al., 2021 [29]	1	1	1	1	1	1	1	1	0	1	0	NA	0	1	NA	NA	0	1	1	0	0	1	1	1	71%
Gedik, 2020 [30]	1	1	1	1	1	1	1	1	0	0	0	NA	0	1	NA	NA	0	0	1	0	0	1	0	0	52%
Jang et al., 2021 [31]	0.5	0.5	1	1	1	1	1	1	0	0	0	NA	0	1	NA	NA	0	1	1	0	1	1	1	1	66%
Khan et al., 2020 [32]	1	1	1	1	1	1	1	1	0	1	0	NA	0	1	NA	NA	0	1	1	0	0	1	1	1	71%
Miethke-Morais et al., 2021 [33]	1	1	1	1	1	1	1	1	0	1	0	NA	0	1	NA	NA	0	1	1	0	1	1	1	1	76%
Ohsfeldt et al., 2021 [34]	1	1	1	1	1	1	1	1	0	0	0	NA	0	1	NA	NA	0	1	1	0	1	1	1	0	67%
Popescu et al., 2022 [35]	1	1	1	1	1	1	1	1	0	0	0	NA	0	1	NA	NA	0	1	1	0	0	1	1	1	67%
Reddy et al., 2021 [36]	1	1	1	1	1	1	1	1	0	1	0	NA	0	1	NA	NA	0	1	1	0	1	1	1	1	76%
Seringa et al., 2022 [37]	1	1	1	1	1	1	1	1	0	0	0	NA	0	1	NA	NA	0	1	1	0	1	1	1	1	71%

Primary Outcomes

A total of six studies examined the daily cost per patient. Among them, three reported the median cost with interquartile range, while five provided the mean cost with standard deviation (Tables 3, 4). Brazil reported the highest mean daily cost per patient ($62,066.28, SD not reported), while Turkey reported the lowest ($236.72 ± 94.45). The highest median cost was found in the USA ($2,962.32, IQR not reported), while the lowest median cost was reported in Romania ($311.34, IQR: $129.60 - $674.66). However, this information was seldom reported; only three studies supplied such data.

**Table 3:** Mean ICU cost per day per patient N = total number of patients admitted to ICU, ICU = intensive care unit, SD = standard deviation, GDP = gross domestic product, N/R = not reported, CI = confidence interval, Currency = US$

Author	Country	N	Mean ICU cost per day per patient	SD	95% CI	GDP
Gedik, 2020 [30]	Turkey	66	236.72	94.45	214 - 260	719,954,821,683.31
Khan et al., 2020 [32]	Saudi Arabia	222	6,724.52	N/R	N/R	703,367,841,222.56
Miethke-Morais et al., 2021 [33]	Brazil	1,683	62,066.28	N/R	N/R	1,448,565,936,739.56
Ohsfeldt et al., 2021 [34]	USA	7,0054	3,660.34	3,355.94	3,630 - 3,690	20,893,743,833,000.00
Reddy et al., 2021 [36]	India	176	889.79	322.54	842 - 937	2,667,687,951,796.56

**Table 4:** ICU daily cost per patient (median and IQR) IQR = interquartile range, GPD = gross domestic product, N/R = not reported, Currency = US$

Author	Country	N patients	Median	IQR	GDP
Ohsfeldt et al., 2021 [34]	USA	70,054	2,962.32	N/R	20,893,743,833,000.00
Popescu et al., 2022 [35]	Romania	36	311.34	129.60, 674.66	284,087,563,695.80
Reddy et al., 2021 [36]	India	176	889.66	674.08, 1,105.59	2,667,687,951,796.56

**Figure 2:**
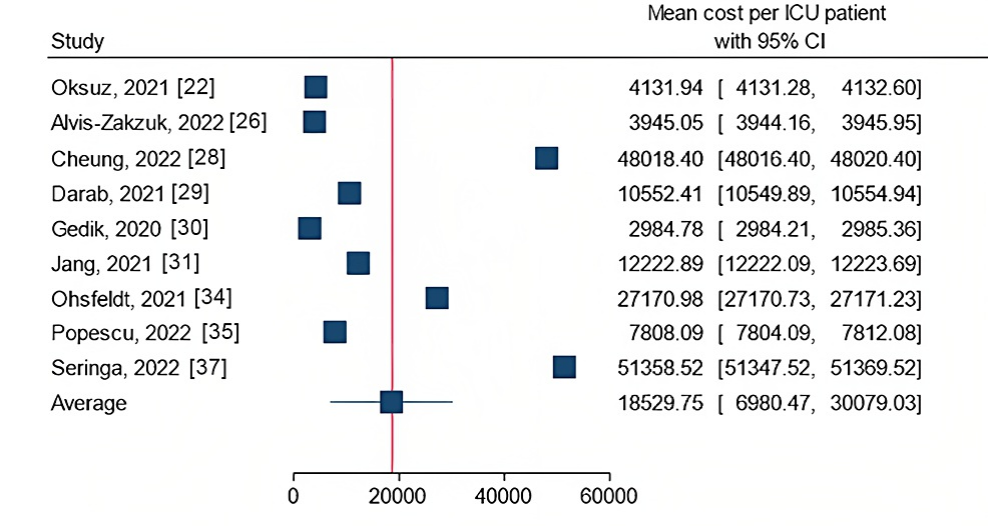
Forest plot of mean costs per ICU patient Oksuz, 2021 [22]; Alvis-Zakzuk, 2022 [26]; Cheung, 2022 [28]; Darab, 2021 [29]; Gedik, 2020 [30]; Jang, 2021 [31]; Ohsfeldt, 2021 [34]; Popescu, 2022 [35]; Seringal, 2022 [37] ICU = intensive care unit, CI = confidence interval, Currency = US$

The most commonly reported outcome was the mean cost per ICU patient. In total, 12 studies provided either the mean or median values for the cost per patient. From nine studies, the average mean cost per ICU patient was determined to be $18,529.75 (95% CI: $6,980.47 - $30,079.03). Figure 2 depicts a forest plot illustrating the mean costs per ICU patient of the studies that reported mean cost with a 95% confidence interval. Portugal had the highest average cost of $51,358.52 (±$30,150.38) per ICU patient, closely followed by Canada with a slight difference of $48,018.40 (±$31,434.29). In contrast, Turkey and Colombia reported the lowest mean costs per patient. Turkey reported $2,984.78 (±$2,395.93) and $4,131.94 (±$4,298.47) in the respective studies, while Colombia reported $3,945.05 (±$2,675.02).

The median values for the total cost per patient were reported by seven studies (Table 5). By far, the highest median value was found in Portugal ($46,414.03, IQR: $31,179.09 - $62,866.68) and the lowest in Turkey with $2,859.19 (Q3-Q1: 3,831.29).

**Table 5:** Total cost per patient (median and IQR) N = total number of patients admitted to ICU, ICU = intensive care unit, IQR = interquartile range, GPD = gross domestic product, N/R = not reported, Currency = US$

Author	Country	N	Median ICU cost per ICU patient	IQR	GDP
Kavalci, 2021 [21]	Turkey	71	14,875.83	15,814.19	719,954,821,683.31
Oksuz et al., 2021 [22]	Turkey	163	2,859.19	3,831.29	719,954,821,683.31
Alvis-Zakzuk et al., 2022 [26]	Colombia	34	4,203.60	2,112.01, 5,568.39	270,299,982,887.01
Ohsfeldt et al., 2021 [34]	USA	70,054	13,722.43	N/R	20,893,743,833,000.00
Popescu et al., 2022 [35]	Romania	36	5,368.89	754.42, 16,367.85	284,087,563,695.80
Reddy et al., 2021 [36]	India	176	10,907.50	6,885.23, 16,626.61	2,667,687,951,796.56
Seringa et al., 2022 [37]	Portugal	29	46,414.03	31,179.09, 62,866.68	228,539,245,045.34

Secondary Outcomes

A total of eight studies reported the mean length of stay in the ICU (Table 6). The Republic of Korea reported the highest mean value of 29.4 days (±7.80), while the lowest was observed in India with nine days (±5.98). Additionally, five studies reported median values of the length of stay, with Turkey having the highest median of 13 days (Q3-Q1: 12), and the lowest observed in the USA with five days (Table 7).

**Table 6:** Length of Stay in ICU (mean and SD) N = total number of patients admitted to ICU, ICU = intensive care unit, LOS = length of stay, SD = standard deviation, CI = confidence interval

Author	Country	N	LOS mean	LOS SD	95% CI
Oksuz et al., 2021 [22]	Turkey	163	14.8	12.00	13 - 16.6
Alvis-Zakzuk et al., 2022 [26]	Colombia	34	10.2	7.20	7.78 - 12.6
Gedik, 2020 [30]	Turkey	66	14.74	13.19	11.6 - 17.9
Jang et al., 2021 [31]	Republic of Korea	846	29.4	17.80	28.2 - 30.6
Ohsfeldt et al., 2021 [34]	USA	88,530	7.7	9.1	7.64 - 7.76
Popescu et al., 2022 [35]	Romania	36	14.1	8.1	11.4 - 16.8
Reddy et al., 2021 [36]	India	176	9	5.98	8.12 - 9.88
Seringa et al., 2022 [37]	Portugal	29	10.1	6.00	7.92 - 12.3

**Table 7:** Length of Stay in ICU (median and IQR) N = total number of patients admitted to ICU, IQR = interquartile range, N/R = not reported

Author	Country	N	LOS median	IQR
Kavalci, 2021 [21]	Turkey	71	11.50	11.00
Oksuz et al., 2021 [22]	Turkey	163	13	12.00
Ohsfeldt et al., 2021 [34]	USA	88,530	5	N/R
Reddy et al., 2021 [36]	India	176	9	5.00 - 13.00
Seringa et al., 2022 [37]	Portugal	29	9	7.00 - 12.00

Six studies from Spain, Canada, Turkey, and Iran, reported mean costs per patient in the general ward, except for the ICU, as shown in Table 8 [22,27-31]. As expected, the costs in the general ward were significantly lower than in the ICU. The lowest mean costs per general ward patient were found in the two studies from Turkey [22,30] and the highest in Canada [28].

**Table 8:** Total costs per patient (mean, SD) ICU vs general ward N = total number of patients, ICU = intensive care unit, LOS = length of stay, SD = standard deviation, N/R = not reported, GDP = gross domestic product, Currency = US$

Author	Country	N patients in ICU	Mean cost per ICU patient (SD)	N Patients in General Ward	Mean cost per general ward patient (SD)	GDP
Oksuz et al., 2021 [22]	Turkey	163	4,131.94 (4,298.47)	893	1,133.26 (838.24)	719,954,821,683.31
Calderón-Moreno et al., 2022 [27]	Spain	283	22,940.63	N/R	6,822.07	1,281,484,640,043.58
Cheung et al., 2022 [28]	Canada	944	48,018.40 (31,434.29)	2.926	14,435.32 (9,400.32)	1,645,423,407,568.36
Darab et al., 2021 [29]	Iran	36	10,552.41 (7,645.00)	441	2,369.22 (2,091.65)	231,547,571,240.47
Gedik, 2020 [30]	Turkey	66	2,984.78 (2,395.93)	393	900.08 (681.18)	719,954,821,683.31
Jang et al., 2021 [31]	Republic of Korea	846	12,222.89 (11,729.86)	6.744	3,404.32 (3,199.14)	1,637,895,802,792.90

**Figure 3:**
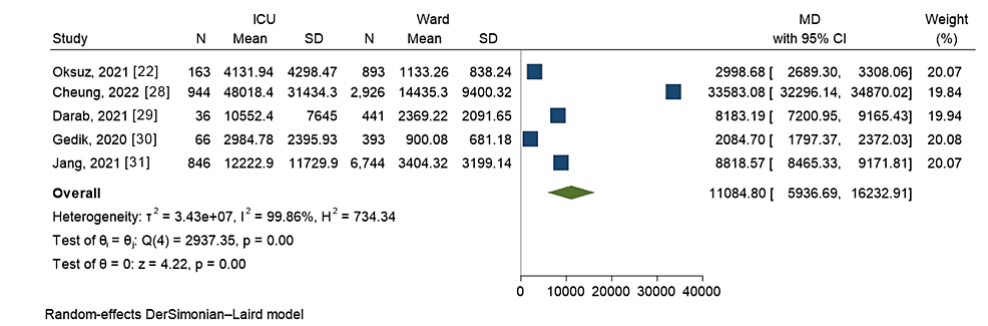
Forest plot of meta-analytical comparison between ward costs and intensive care costs Oksuz, 2021 [22]; Cheung, 2022 [28]; Darab, 2021 [29]; Gedik, 2020 [30]; Jang, 2021 [31] ICU = Intensive Care Unit, SD = Standard deviation, N = Total number of patients, CI = Confidence interval, MD = Mean difference, Currency = US$

For the outcome of mean cost per patient, a meta-analytical comparison between ward costs and intensive care costs was conducted using data from five studies. The meta-analysis revealed an overall mean difference of $11,085 (p<0.001) in favor of the ICU over the ward (Figure 3). This indicates that the mean costs per ICU patient were significantly higher compared to the mean costs per ward patient.

Discussion

This systematic review synthetizes the available evidence relating to the costs of ICU hospitalization due to COVID-19 without any country limitation. This is the first publication of a systematic review of the economic burden for ICU hospitalized COVID-19 patients. The economic impact of COVID-19 in terms of cost was reported as significant in all studies reviewed. Our analysis showed that there are large cost differences between the studies reviewed. Despite a thorough examination of the data, this discrepancy in the cost estimates could not be readily explained, particularly for countries with similar characteristics.

When comparing the medical costs, we observed remarkably low costs in three studies conducted in Turkey. The findings from these Turkish studies [21,22,30] diverged significantly from the other economic evidence. It is important to note that as of September 25, 2022, Turkey officially reported 16,873,793 cases of coronavirus and 101,139 deaths [38]. In comparison, Germany, with a similar population (84,680,273 for Turkey and 83,695,430 for Germany), reported 32,952,050 cases (51.20% more) and 149,458 deaths (67% more). We also noticed that the total expenditure on health per capita in Germany was $6,503.36, while in Turkey, this figure was substantially lower at approximately $395.24 [17]. These findings align with the criticism of Turkey's handling of the coronavirus as well as its reporting policy [39].

It is also important to consider each country's GDP, which represents the monetary value of all goods and services produced in a year. It serves as an indicator of the size of a country's economy [40]. Taking this into account, it can be argued that GDP is a valuable tool for understanding economic factors. For instance, Brazil, with a GDP approximately 14 times lower than that of the US, reported average intensive care costs per day per patient that were 16.95 times higher. This stark contrast highlights the economic disparities and challenges faced by Brazil during the crisis. Indeed, studies have indicated that Brazil mishandled the COVID-19 crisis, lacking effective national policies on social distancing guidelines and facing issues with the vaccination process. Additionally, government officials disseminated false information regarding the origin and severity of the disease [41,42]. These factors contributed to the misallocation of resources and the need for significant expenditures to address the emergency situation [43]. Furthermore, Brazil experienced a significant currency depreciation (27.2% from March 11 to May 14, 2020) and subsequent exchange rate volatility when compared to other emerging economies [43].

Similarly, Korea reported a relatively high mean cost per patient in the ICU ($12,222.89 ± 11,729.86). However, a recent study compared ICU costs in Korea from 2010 to 2019 and found that the mean total ICU cost per patient was approximately $6,370.00 in 2010 and $11,131.50 in 2019 [44]. In contrast, Canada, which has a similar GDP to Korea (Canada: $1,645,423,407,568.36 vs. Korea: $1,637,895,802,792.90), reported a much higher average cost per patient in the ICU of $48,018.40 (±31,434.29). Since the outbreak began, the Korean government has taken immediate measures to minimize the spread of the virus and provide free health services to low-income groups. Despite having the second-highest number of cases after China in February and early March, Korea successfully contained the transmission of the virus without implementing a national lockdown. This achievement was made possible because Korean citizens actively followed quarantine guidelines. The previous experience of Korea in handling similar outbreaks, such as the Middle East respiratory syndrome (MERS) outbreak in 2015, demonstrates the importance of early response and adopting a systematic approach with the necessary procedures [45].

Iran, Colombia, and Portugal have similar GDPs, but Portugal reported the highest mean cost per ICU patient (approximately $51,358.52) compared to these countries. Iran, on the other hand, reported a mean cost of $10,552.41, which is consistent with another study conducted in Iran that reported relatively high direct and indirect healthcare costs for COVID-19 patients [46].

In addition, two studies in Turkey reported a similar ICU length of stay (LOS) of approximately 14.77 days (14.74 ± 13.19 and 14.8 ± 12.00). However, another study conducted in 2019 reported a shorter LOS in ICU in Turkey, averaging 10.99 ± 12.6 days. This increase in hospitalization days implies an increase in hospitalization costs [22,30,47]. Furthermore, the one study from Portugal included in our review reported a median ICU LOS value of nine days (7.00 - 12.00), while another study examining the period from January 2015 to June 2019 in Portugal reported a median ICU LOS value of four days (2.00 - 9.00) [37,48]. The study from India included in our analysis reported a mean ICU LOS value of nine days (IQR: 5-13). However, a previous study on the length of stay in Indian hospitals in 2014 reported an average ICU stay of four days, with the minimum being one day and the maximum being 11 days [36,49]. The observed increase in the number of days of hospitalization for patients with COVID-19 in the ICU has significant implications for the total cost per admission. When patients require an extended duration of stay in the ICU, it necessitates the provision of intensive medical care, specialized equipment, and round-the-clock monitoring by healthcare professionals. These additional resources and services contribute to the overall cost incurred during the patient's hospitalization.

Undoubtedly, the ICUs incur higher costs compared to general wards, as strongly supported by our evaluation. The results from six studies consistently demonstrated significantly higher mean costs per patient in the ICUs compared to the corresponding costs in general wards. Apart from Turkey [22,30], Iran [29] reported ICU costs four times higher than those in general wards ($10,552.41 vs. $2,369.22, respectively). Additionally, Korea, with a GDP similar to that of Iran, reported a mean cost of $3,404.32 per patient in the general ward. Among these six studies, the highest mean cost per patient in the general ward was found in Canada, amounting to $14,435.32, which was three times lower than the corresponding cost in the ICU. However, it is important to note that the differences in reported costs between the studies included in our review can be partially explained by the lack of standardization in costing methods. Notably, we also observed inconsistencies in the presentation of important components of case management costs across studies. This lack of consistency is a common issue in systematic reviews of economic analyses, which often makes meaningful comparisons challenging, to say the least. Different pricing for the same technology across borders is to be expected due to variations in tax rates, transportation costs, procurement practices, intellectual property, and patents [50]. Overall, the quality of the included studies was generally low, with only two studies standing out as exceptions. The low quality of the reports can be attributed to weak cost analysis methodologies and practices. It is crucial for future studies to improve the rigor and standardization of economic evaluations in order to provide more reliable and comparable cost estimates. This will enhance our understanding of the economic burden associated with ICU care for COVID-19 patients and facilitate better decision-making in healthcare resource allocation.

Finally, to obtain a comprehensive understanding of the economic burden caused by COVID-19, it is crucial to consider and calculate the indirect and intangible costs associated with the disease. It is very important for researchers to report what the costs consist of because it allows healthcare providers to make informed decisions about medical treatments, procedures, and services. Without transparency regarding the specific costs involved, a vague and incomplete picture may emerge. By reporting on the specific cost components, hospitals can also identify areas where expenses can be reduced or operations can be optimized. This understanding of direct costs allows hospital administrators to make informed resource allocation decisions, negotiate pricing with suppliers, and streamline processes, ultimately improving efficiency and the quality of care. Furthermore, reporting on costs can aid in compliance with regulatory requirements, such as insurance billing and reimbursement policies. Accurate reporting on direct costs helps hospitals ensure that they adhere to industry standards and avoid potential legal and financial consequences. Transparent reporting on cost components promotes accountability, builds trust with patients and healthcare providers, and ultimately leads to better quality and more affordable healthcare.

It is worth mentioning that there are methodological challenges, including the exclusive use of the arithmetic mean by many researchers to describe the data. Given the likelihood of a positively skewed distribution of cost data in many, if not all, of the studies considered, the median (along with the interquartile range) would have provided a more informative measure of the average cost per person and a better description of the data distribution [51]. Particularly in the context of COVID-19, the mean and confidence interval are useful summary statistics for policymakers who seek to understand the total cost of the disease for a cohort of patients as a whole. Policymakers need to practically evaluate the total cost of each treatment delivered, highlighting the importance of considering the mean and confidence interval [52].

Discrepancies in the reported costs of COVID-19 can also be attributed to the lack of well-defined guidelines for conducting cost-of-illness studies, making comparisons between studies extremely difficult. This challenge arises when researchers provide insufficient data on costs and the sources used, thereby hindering the assessment of the reliability and validity of the reported costs. Furthermore, as any cost estimate inherently contains a degree of uncertainty, inaccuracy, and ambiguity, it is crucial for researchers to test the sensitivity of their results through sensitivity analyses, where different assumptions and/or estimates are utilized [53]. None of the 14 studies reviewed in our study considered these assumptions by means of sensitivity analyses.

Outbreaks of infectious diseases can have a devastating impact on society, both in terms of human lives lost and economic costs. The COVID-19 pandemic, for example, has had an enormous impact on health systems, economies, and daily life around the world. Countries should have been better prepared for an outbreak because infectious diseases can spread quickly and have devastating consequences. The COVID-19 pandemic has highlighted the need for countries to have robust public health infrastructure and preparedness plans. A lack of preparedness can result in delayed responses, inadequate resources, and increased morbidity and mortality. Being prepared means having the necessary equipment, trained personnel, and systems in place to respond quickly and effectively to an outbreak. A pre-plan is crucial because it enables countries to respond to an outbreak in a coordinated and efficient manner. A pre-plan should outline the steps that need to be taken in the event of an outbreak, including surveillance, contact tracing, isolation, quarantine, and vaccination programs. It should also identify the stakeholders involved, their roles and responsibilities, and the resources required. By having a pre-plan in place, countries can respond more quickly and effectively, minimize the spread of the disease, and reduce its impact on society. A better health policy for dealing with a new outbreak should be based on several key principles [54]. First, it should prioritize public health and preparedness. This means investing in public health infrastructure, such as diagnostic testing, contact tracing, and vaccination programs, and ensuring that there are enough trained personnel and resources to respond effectively. Second, it should prioritize the protection of essential workers, including healthcare workers, first responders, and other frontline workers. This includes providing personal protective equipment, hazard pay, and other forms of support. Third, it should prioritize equity and address the needs of vulnerable populations, such as the elderly, the immunocompromised, and those living in low-income communities. This may include targeted outreach and support programs to ensure that they have access to necessary resources. Investing in preparedness may be more economically efficient in the long run than waiting for an outbreak to occur. Outbreaks can have a significant impact on economies, as they can result in disruptions to supply chains, decreased consumer confidence, and reduced economic activity [55]. By investing in preparedness, countries can potentially avoid these impacts and limit the economic costs of an outbreak. Additionally, preparedness may help to reduce healthcare costs, as prompt and effective responses can limit the spread of the disease and minimize the need for costly interventions such as hospitalizations and intensive care [56].

Limitations

This study has several limitations. Our systematic review was limited to English-language journal articles, and therefore, a publication bias should not be neglected, although no geographic restriction was applied. The marked heterogeneity and uniformity of reported data between studies led to significantly different results and made it difficult to compare data to estimate pooled results for individual countries or regions. Adding to this, there are few that have studied hospital costs due to COVID-19, and most of them have either dealt with the general ward or have not separated the costs of the general ward and the intensive care unit.

Another limitation stems from the fact that no study used a proper cost-effectiveness analysis, such as quality-adjusted life years (QALY), to express results in clinical terms without ignoring the possible misclassification of costs that are (or are not) attributable to COVID-19. For example, we were unable to determine from the relevant literature whether hospitalizations were actually related to COVID-19. Our results revealed a marked heterogeneity of evidence, which prevents us from drawing firm conclusions about the current cost burden. Therefore, any generalization of the results at a global level should be treated with caution.

## Conclusions

COVID-19 has had a profound impact on healthcare systems and countries worldwide, imposing a substantial economic burden regardless of the specific direct costs involved. Studies included in our review have revealed methodological disparities and significant variations in cost assessments, which pose challenges in comparing and analyzing them effectively. In particular, the economic implications of COVID-19 on ICUs have not been extensively explored or well-documented. Conducting further studies to estimate the costs associated with COVID-19 could yield more comprehensive and reliable data, enabling healthcare providers and policymakers to make well-informed decisions. By obtaining clearer insights into healthcare costs, stakeholders can enhance the organization of patient care, striving for maximum efficiency in resource allocation and management.
